# Bioinspired Metal–Polyphenol Materials: Self-Healing and Beyond

**DOI:** 10.3390/biomimetics4020030

**Published:** 2019-04-04

**Authors:** Amanda Andersen, Yaqing Chen, Henrik Birkedal

**Affiliations:** Department of Chemistry and iNANO, Aarhus University, 14 Gustav Wieds Vej, 8000 Aarhus, Denmark; amandaandersen@inano.au.dk (A.A.); ychen@inano.au.dk (Y.C.)

**Keywords:** coordination chemistry, catechols, mussel-inspired, self-healing, phenols, tannins, DOPA, multifunctional materials

## Abstract

The blue mussel incorporates the polyphenolic amino acid l-3,4-dihydroxyphenylalanine (DOPA) to achieve self-healing, pH-responsiveness, and impressive underwater adhesion in the byssus threads that ensure the survival of the animal. This is achieved by a pH-dependent and versatile reaction chemistry of polyphenols, including both physical interactions as well as reversible and irreversible chemical bonding. With a short introduction to the biological background, we here review the latest advances in the development of smart materials based on the metal-chelating capabilities of polyphenols. We focus on new ways of utilizing the polyphenolic properties, including studies on the modifications of the nearby chemical environment (on and near the polyphenolic moiety) and on the incorporation of polyphenols into untraditional materials.

## 1. Introduction

There is a significant current focus on approaches to design and harness smart materials employing chemical interactions in new ways to achieve advanced functions. One rapidly developing area is the use of the diverse chemistry of polyphenols, such as catechols (dopamine, l-3,4-dihydroxyphenylalanine (DOPA), etc.) or pyrogallols (tannic acid (TA), etc.), which originates from the fascinating adhesive and self-healing capabilities of mussel byssus threads. In this review, we focus on how the metal-chelating capabilities of these moieties can be used for making smart materials. We start with a brief discussion of the biological source of inspiration and general catechol chemistry, and end by providing examples of how this chemistry can be extended and harnessed in materials design.

### 1.1. The Blue Mussel Byssus

**Scheme 1:**
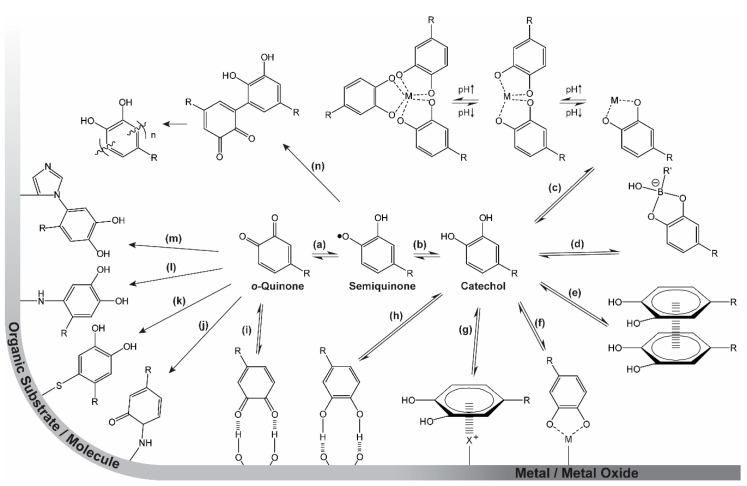
The versatility of catechol chemistry. (**a**) Reversible reduction (+1e^−^, +1H^+^)/oxidation (−1e^-^, −1H^+^) from/to *o*-quinone to/from semiquinone. (**b**) Reversible reduction (+1e^−^, +1H^+^)/oxidation (−1e^-^, −1H^+^) from/to semiquinone to/from catechol. (**c**) Reversible, pH-dependent coordination of free metal (M) ions. (**d**) Reversible formation of catechol–boronate species. (**e**) π–π stacking with other aromatic moieties. (**f**) Reversible adhesion to inorganic surfaces by coordination bonds. (**g**) π–cation interactions with inorganic cationic species (i.e., metals) or organic cationic species (i.e., positively charged amino acids (X^+^)). (**h**) Hydrogen bonding to surfaces or molecules as catechol/donor or (**i**) *o*-quinone/acceptor. (**j**) Irreversible bonding to organic molecules or surfaces by Schiff base reactions or (**k**) Michael-type additions with thiols, (**l**) amines, and/or (**m**) other catechols yielding (**n**) dimers and eventually polymers. R represents the remainder of the catechol-containing molecule.

The byssus threads of the blue mussel (*Mytilus* sp.) provide strong adhesion between the soft tissue of the mussel and various substrates in both wet and dry conditions [1,2,3,4]. The completion of this task alone is remarkable, but furthermore, the byssus threads possess a coating that can heal after damage, even though no cells are present [1,5,6,7]. These abilities are desired for a wide range of materials, for example within the fields of adhesive and biomedical materials. Therefore, the chemical structures and mechanisms related to these functions have been extensively investigated [2,3,4,5,6,8,9,10,11,12,13,14,15,16]. Since the identification of the amino acid sequences and spatial distribution of the mussel foot proteins (mfps), the field of materials inspired by the mussel byssus has grown considerably. The proteins mfp-3 and mfp-5, constituents of the mussel byssus adhesive pad and the byssus cuticle, have high contents of the amino acids lysine and post-translationally modified DOPA (Scheme 1, R = CH_2_CH(NH_2_)COOH), as well as ferric ions [4,5,6,8,9,11,13]. It should be stressed that many questions remain unanswered as to the exact mechanisms acting in the different parts of the byssus and its formation, including synergies between different chemical moieties within each protein and between proteins as well as the process and kinetics of delivery [17]. However, the high amount of possible reversible interactions of DOPA (Scheme 1, right), especially the coordination bonds between DOPA and Fe(III) (Scheme 1c), are believed to enable the self-healing properties of the byssus cuticle [18]. Additionally, the many possible reaction paths displayed in Scheme 1 are responsible for the adhesion to different surfaces and cross-linking of the protein by reaction with other moieties along the protein [19]. These interactions highlight the role of DOPA, but it should be stressed that the complex nature of the mfps strongly suggests that multiple counterbalancing interactions, whose relative roles remain to be fully understood, are at play.

### 1.2. pH-Dependent Material Behavior

The chemistry, mechanisms, and synergistic effects of these moieties, especially the DOPA residue (Section 1.2, Scheme 1), have been heavily investigated in both native proteins and several model systems [2,3,4,5,6,8,9,10,11,12,13,14,15,16]. The chemistry displayed in Scheme 1 is heavily dependent on pH: Firstly, the capabilities of the aromatic ring and hydroxyl groups to participate in various physical interactions are affected by pH because the catechol is a weak acid. Furthermore, at low pH, the catechol form is favored, whereas at higher pH, oxidation to the *o*-quinone (facilitating covalent bonding) leads to cross-linking. The metal-binding capability is also highly pH dependent. At low pH, only non-cross-linking (mono-) coordination is possible, while at increased pH, higher-order (bis- and tris-) coordination of the catechol results in protein cross-linking. This pH-dependent chemistry is utilized by the mussel both in establishing strong adhesion in the pad [5] and in the fabrication of the protective self-healing cuticle of the byssus thread [8]. Inspired by this chemistry, researchers have demonstrated the potential for its use in various materials constructs, including self-healing gels and coatings, as well as strong adhesives [10,11,12,15,16]. The multifaceted chemistry of catechols makes them very interesting and useful tools for materials design [11,20,21,22,23]. Herein, we especially focus on the metal-binding capability of catechols and catechol analogues, and its use in designing self-healing materials.

## 2. Catechol Chemistry

The diverse chemistry of catechols is summarized in Scheme 1. Strong, yet reversible, and very pH-dependent coordination bonds establish strong adhesion to inorganic surfaces (metals and metal oxides) [1,24,25,26,27] (Scheme 1f,g) as well as hard metal ions in solution [6,28,29,30,31,32,33,34] (Scheme 1c). Similarly, catechols can participate in boronate species (Scheme 1d) that are reversible, even if they possess a high degree of covalency [35,36,37,38]. By oxidation to the *o*-quinone form (Scheme 1b + a), the moiety becomes highly reactive towards a wide range of organic species (surface-bound or in solution), resulting in covalent linkages [14,23,39]. Reactions include Schiff base reactions (Scheme 1j) and Michael-type additions with a variety of amines (Scheme 1l,m), thiols (Scheme 1k), and other catechols (Scheme 1n) (resulting, e.g., in polymerization of dopamine to give polydopamine) [23,40]. Furthermore, the aromatic π-system enables the catechol and/or *o*-quinone moieties to interact with each other by π–π stacking [41,42] (Scheme 1e) or with cations on surfaces or in solution by π–cation interactions [26,43]. Finally, both the catechol and *o*-quinone have the ability to participate in hydrogen bonding (Scheme 1h,i) [44].

### 2.1. Catechol Coordination Chemistry

**Figure 1:**
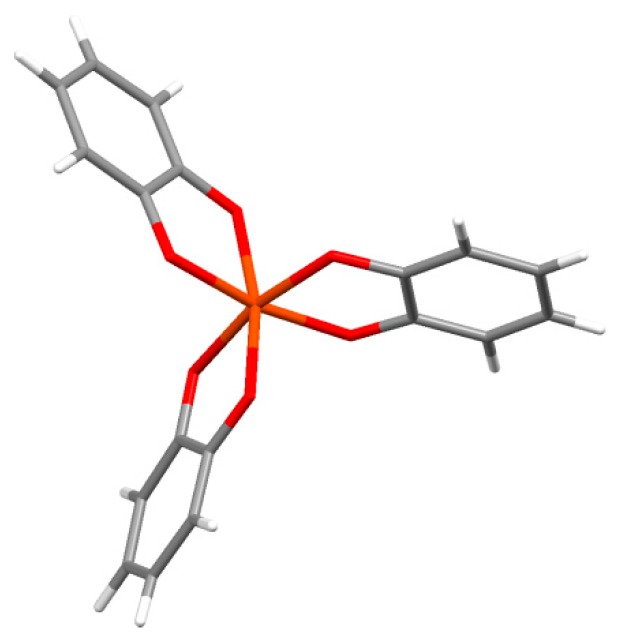
Coordination geometry of the tris(catecholato)Fe(III) complex from the crystal structure of piperidinium tris(pyrocatecholato)ferrate(II) sesquihydrate [45].

Catechols form coordination complexes with a wide range of metal ions, and form tris-complexes at high pH. Several of these have been crystallographically examined, as illustrated in Figure 1, which shows part of the crystal structure of piperidinium tris(pyrocatecholato)ferrate(II) sesquihydrate [45]. The charge of the complex depends on the metal oxidation state and the ligand charges. For example, the tris-complex of iron or aluminum (or other group III metals) in oxidation state III will have a charge of −3. Therefore, such complexes require local counterions. For the iron complex in Figure 1, piperidenium ions are present. We speculate that this requirement of local charge neutralization may be an additional role played by the many amines from lysine in the cuticle mfp-1 protein in the mussel byssus thread.

### 2.2. Strengths of Catechol Metal Binding

Several materials inspired by the utilization of the versatile chemistry of DOPA have been developed (see Section 3 and Section 4). Many of these are polymeric constructs with catechols incorporated along the backbone. Incorporation of different metal ions and mfp-mimicking moieties, such as amines, has also been investigated, based on the notion that the chemical environment of the catechol affects its reaction chemistry [32,34,46,47,48,49,50,51].

**Figure 2:**
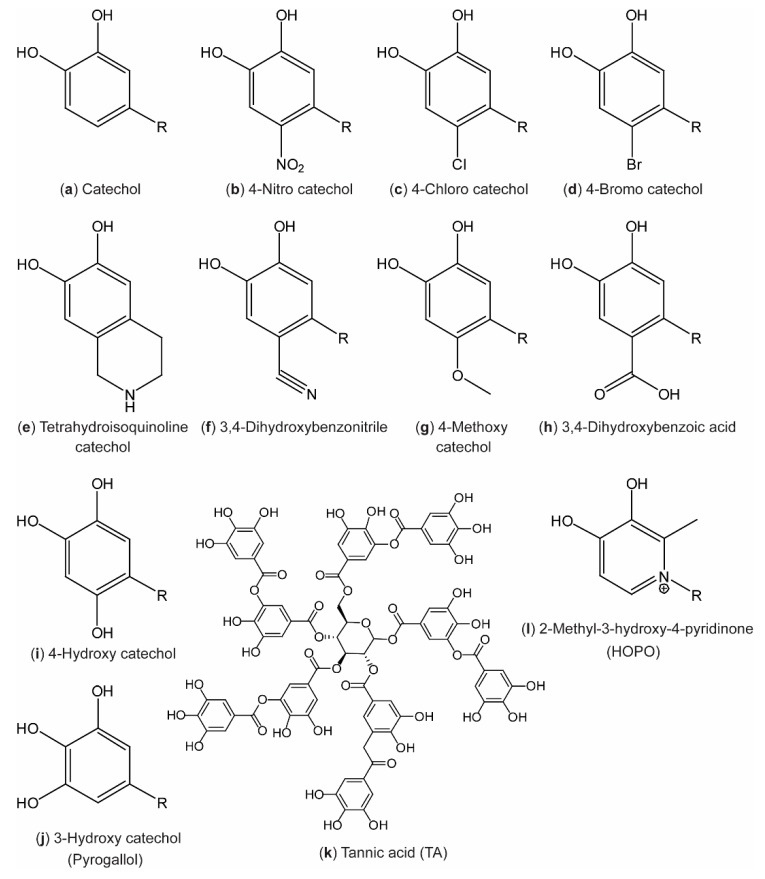
Catechol analogues reported in the literature. The molecules have been named according to the International Union of Pure and Applied Chemistry (IUPAC) order of precedence, assuming that the R group (representing the remainder of the molecule) has lower priority than the catechol hydroxyl groups.

More recent designs seek to dictate the behavior of the resulting materials by substitution of electron- withdrawing or -donating groups onto the aromatic ring (Figure 2b–k) and even in the aromatic ring (Figure 2l). Such designs are discussed in Section 4.

Recently, the interest in understanding the contribution/influence of the roles of the different interactions displayed in Scheme 1, as well as the nearby chemical environment, has increased. Several studies probing the strength and importance of the different catechol interactions have been published, both in bulk materials and for single molecules, revealing new pieces in the puzzle of catechol-based adhesion.

**Figure 3:**
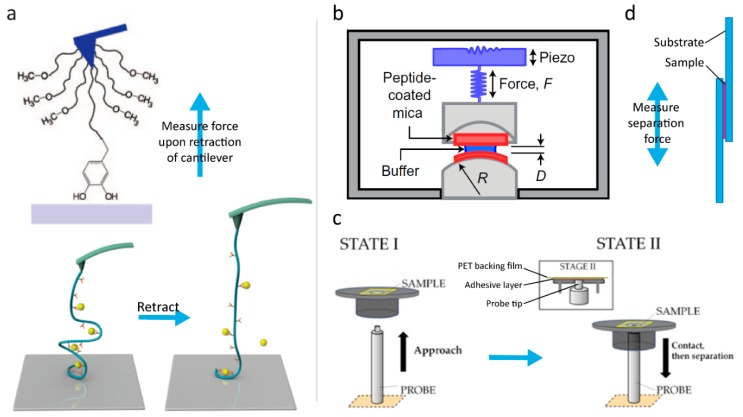
Methods of evaluating bonding strength. (**a**) Atomic force spectroscopy used for probing the strength of single-molecule adhesion (top) and catecholato–Fe(III) coordination bonds cross-linking a polymer (bottom). The sample is fixed on a cantilever and the strength is evaluated by measuring the force needed to retract the Sciences of the USA. (Bottom) Reprinted (adapted) with permission from [33]. Copyright 2017 American Chemical Society. (**b**) Set-up of a surface forces apparatus: The sample is fixed on mica surfaces that are brought in contact, and the separation force is measured. Reprinted by permission from MacMillan Publishers Ltd. [27], Copyright 2017. (**c**) Set-up of a Probe Tack device: The sample is fixed on a backing film (here polyethylene terephthalate (PET)) and brought in contact with a probe, which is then retracted and the adhesion force evaluated. Reprinted (adapted) with permission from [53]. Copyright 2016 American Chemical Society. (**d**) Lap-shear test: The sample is fixed between two pieces of overlapping substrate and the adhesion force is measured when separating the substrates in the direction indicated by the arrow.

In 2006, Lee et al. [1] pioneered investigations of the strength of catechol–surface interactions in a breakthrough paper on single-molecule force spectroscopy (Figure 3a, top). In this study, the catechol–TiO_2_ coordination bond was estimated to have a strength corresponding to about half a typical covalent bond. Recently, using similar methodology, Das et al. [25] reported on a comprehensive study in which single-molecule force spectroscopy measurements had been performed on short, amino acid-terminated poly(ethylene glycol) (PEG) strings on TiO_2_ surfaces under neutral and slightly alkaline conditions. At neutral pH (7.2), the authors found that the adhesion forces for Phe and Tyr were similar (69 ± 19 and 89 ± 24 pN, respectively), whereas the introduction of a second hydroxyl group in DOPA increased the force significantly (383 ± 21 pN). This underlines the importance of the bidentate coordination of the catechol when adhering to inorganic surfaces. Furthermore, the authors found that further substitution of a third hydroxyl group on the fourth position in the aromatic ring (4-hydroxy catechol, Figure 2i) led to a further increase (422 ± 34 pN). However, when substituting a nitro group on the same position (4-nitro catechol, Figure 2b), the behavior shifted to a bimodal distribution, suggesting that two different bonding events can occur. A similar bimodal distribution was also observed for DOPA and 4-hydroxy catechol at alkaline pH (9.8), with one distribution corresponding to the adhesion force value at neutral pH, while the other was centered around a significantly lower force. This behavior is consistent with single-molecule fluctuations between the catechol and *o*-quinone form (Scheme 1a,b), with the two having different affinities towards the surface (high for catechol and low for semiquinone). The observation that the 4-nitro catechol gives rise to a bimodal distribution at lower pH can be ascribed to the significant lowering in p*K*_a1_ of the catechol, as described in Section 4.

Interestingly, the finding that in single-molecule experiments, the adhesion force goes DOPA > Tyr > Phe is in contrast to the conclusions of Gebbie et al. [26]. In this work, peptides with a high Lys content and high content of Phe, Tyr, DOPA, or Leu (control) were synthesized, and their relative adhesion performances were evaluated by compression and separation between two mica substrates in a surface forces apparatus (SFA) (Figure 3b) at pH 2.5. They reported that the work of adhesion in DOPA- and Tyr-containing peptides is similar (3.6 ± 0.4 and 4.0 ± 0.6 mJ m^−2^, respectively), while that of Phe peptides is significantly higher (10 ± 3 mJ m^−2^). However, the measured failure probed here is in the bulk of the materials (i.e., cohesive): The superior performance of Phe compared to DOPA and Tyr is ascribed to the stronger interaction of the π–cation interactions for saturated aromatic rings than for phenol-substituted ones [26,52]. We note also that the difference in pH indicates that Gebbie et al. [26] worked purely with molecules in the catechol form, whereas the higher pH in the work of Das et al. [25] allowed deprotonation to facilitate coordination and also provided surfaces with active coordination sites.

More interestingly, Clancy et al. reported no significant differences in peptides containing catechol or benzyl groups (and different ratios of cationic and hydrophobic residues), neither in wet (salt/no salt, pH 3, 7, 9, and 11) nor dry adhesion, when probing adhesion forces in a PT-1000 Polyken^TM^ Probe Tack device on Mylar^®^ polyethylene terephthalate (PET) sheets (Figure 3c) [53]. They did, however, find a correlation between the adhesion force and the ratio of cationic to hydrophobic residues in the remainder of the peptide. This is in agreement with the work of Mu et al. [27], who investigated the influence of polymer backbone polarity on catechol adhesion by introducing a catecholic moiety at ≈20% of the monomers in polystyrene, poly(acrylate-co-acrylamide), polyacrylamide, and poly(*N*-vinylpyrrolidone), and comparing wet (seawater) and dry adhesion using a lap-shear test (Figure 3d) on aluminum, glass, and polytetraflourethylen substrates. They found that the bonding strength increased with increasing polarity of the backbone polymer, demonstrating the importance of the chemical environment for catechol adhesion. However, as pointed out by the authors, this conclusion did not factor in the difference in hydrophilicity of the polymers or the synergistic effects of the adjacent monomers that have been proven to affect adhesion [54,55], especially when amines are present [54].

In 2017, Li et al. [33] probed the strength of the catecholato–Fe(III) coordination bond using atomic force spectroscopy (Figure 3a, bottom) by stretching single chains of catechol-containing polymers cross-linked with Fe(III) ions. At neutral pH (7.2), a bimodal distribution, with peaks at ≈100 and ≈200 pN, was found and assigned to the tris- and bis-complexes (Scheme 1c), respectively. At pH 9.7, a single distribution at ≈100 pN was observed and assigned to a contribution from only the tris-complex, which was thus concluded to be weaker than the bis-complex. This finding may seem to be in contradiction to the findings of several hydrogel studies showing that the stiffness of hydrogels cross-linked by metal–catechol coordination becomes stiffer at higher pH (and therefore higher coordination of the metal) [32,34]. However, this gel stiffness results from increased cross-linking density, so increased stiffness can be obtained even if each individual bond is not stronger.

Altogether, the abovementioned studies underline the contributions of the various catechol interactions to the cross-linking/strength of materials.

## 3. Self-Healing Materials Based on DOPA

The metal-binding capability of catechols has been harnessed in several materials since the initial work of Holten-Andersen et al. [34] that showed how to incorporate this chemistry in practice. We reviewed the field until about 2016 elsewhere [10]; therefore, in the following, we will highlight advances made since then. We have chosen to describe briefly the breadth of the field and then present three representative types of contributions in detail (not detracting from the many other excellent works cited).

In 2016, the group of Zeng explored the self-healing mechanism of mussel foot proteins (mfp-1) by studying the reversible iron-mediated bridging between catechols of [Fe(DOPA)_3_]^3−^ by computational methods. They confirmed that the self-healing process achieved through the iron-mediated bridging interaction is a thermodynamically spontaneous process and can be effectively facilitated by hydrogen bond interactions between [Fe(DOPA)_3_]^3−^ and water [56]. Yang et al. [57] used SFA to investigate other key mussel adhesive proteins mfp-3 (subtype mfp-3F) and mfp-5, presenting strong surface adhesion and high DOPA content (approximately 20 and 25 mol %, respectively). They concluded that a high DOPA content in proteins can increase both adhesion and cohesion, but not simultaneously, because in their studies, cohesion was mainly obtained through oxidative cross-linking, while adhesion resulted from catechol interacting with the mica substrate. Based on these results, the authors proposed that mussels may switch the functionality of DOPA at the plaque/substrate interface from surface adhesion to cohesion in response to the microenvironment. Byette et al. [58] treated mussel byssus-derived materials, prepared from a byssus protein hydrolysate, with different metal ions (Na(I), Ca(II), and Fe(III)). They found that the formed metal–ligand interactions and sacrificial bonds, especially between amino acid ligands and Fe(III) ions, effectively increased the stiffness and strength of the materials without sacrificing the extensibility. Following these works, several other contributions utilizing the catechol–metal complexation as underwater self-healing adhesives have emerged [59,60,61,62,63,64,65,66,67,68].

**Figure 4:**
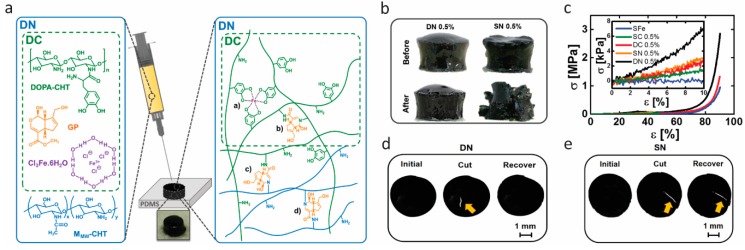
Double cross-link/double network hydrogels with high toughness based on catechol coordination and genipin-mediated covalent cross-links of chitosan. (**a**) Illustration of the concept leading to double cross-link (DC) and double network (DN) hydrogels. (**b**,**c**) Robustness of hydrogels: (**b**) Images of two different hydrogels cross-linked with 0.5% (w/v) genipin:DN (double network of genipin and coordination bonds as displayed in (**a**)) and 0.5% (w/v) genipin:SN (single network of genipin cross-linked chitosan) before and after compression at 50% strain. (**c**) Typical compressive stress–strain curves of different hydrogels with 0.5% (w/v) genipin. (**d**,**e**) Self-healing test of hydrogels: (**d**) Images of DN and (**e**) SN hydrogels before being cut with a blade (initial) , cut (yellow arrow), and after resting for 5 min (recover). Reprinted from [51] by permission of John Wiley & Sons, Inc.

However, self-healing hydrogels with good mechanical behavior are hard to achieve, due to the weak and reversible nature of dynamic interactions of catechol–metal complexation. In 2017, Azevedo et al. [69] developed hydrogels that combined competitive compressive strength, stiffness, toughness, fast recovery, self-healing, injectability, and cytocompatibility for the first time. They employed a double network (DN) strategy, a concept of binary structure whose first network structure is stiff and brittle and the second one is soft and ductile, to enhance the mechanical performance of the developed hydrogels. The double network was composed of DOPA–chitosan (CHT) (i.e., DOPA grafted to chitosan), genipin (GP), Fe(III) ions, and a nonfunctionalized chitosan, as shown in Figure 4a. It contained one network of DOPA–CHT with double cross-links (DC) of covalent bonding (mediated by GP) and catechol–Fe(III) coordinated bonding, and another network of CHT with only covalent cross-links and additional covalent cross-links between the two networks. After application of uniaxial compression at 50% strain, as shown in Figure 4b, the DN hydrogel remained intact, while the single network (SN) (cross-linked only through covalent bonds) was broken into fragments, showing that the double network strategy gave more resilient gels. Figure 4c shows the stress−strain curves of the resultant hydrogels, assessed by applying a uniaxial compression assay. It was apparent that the DN hydrogels exhibited the most robust and ductile properties. They reached 2.46 ± 0.34 MPa in compressive strength, which is more than two-fold of DC, and did not fracture until 90% of deformation due to the synergistic effect of individual networks. The enhanced mechanical properties were achieved by both introducing an energy dissipation mechanism and adjusting the stoichiometry of catechol–Fe(III) coordination by pH value. The self-healing effect is shown in Figure 4d and Figure 3e. The hydrogels were cleaved into two pieces and brought into contact immediately. The DN hydrogel recovered completely, while the SN hydrogel still showed a scar after 5 min. Dynamic oscillatory rheology was further used to determine the self-healing behavior quantitatively. The self-healing was confirmed to result from the dynamic nature of catechol–Fe(III) coordination bonding. However, it was also found that the recovery process of DN hydrogels was delayed due to the reduced mobility of the DOPA–CHT chains resulting from the covalent cross-links in DN. The biomedical potential of these gels was explored by using a double-syringe system as to avoid the drawbacks of invasive implantation techniques, which was possible due to excellent injectability properties, and by testing the cytocompatibility. Thus, an ultratough DN self-healing hydrogel was achieved in a simple way, combined with a set of prominent features, including improved compressive strength and stiffness, injectability, swelling, and cytocompatibility.

**Figure 5:**
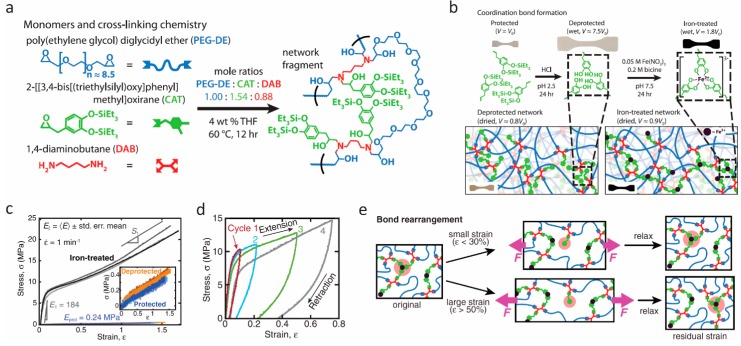
Self-healing elastomers. (**a**) The monomers and cross-linking chemistry. (**b**) Schematics of coordination bond formation following protected network, deprotected network, and ion-treated network. (**c**) Stress–strain curves in uniaxial tensile tests. (**d**) Sequential loading–unloading cycles with rest intervals of 30 min. (**e**) Proposed mechanism for the recovery process based on bond rearrangements. From Filippidi et al. [70]. Reprinted with permission from AAAS.

Although many research groups, including ourselves, have been developing robust self-healing hydrogels, the mechanical performance of these wet, soft systems remains limited, because the abundant water content can weaken physical interactions such as Coulombic attraction, interchain friction, and coordination bonding, leading to a softening of these hydrated materials. To allow mussel-inspired self-healing to be incorporated in a broader suite of technologies, Filippidi et al. [70] designed a toughening elastomer by incorporating reversible catechol–Fe(III) coordination into a dry epoxy network with loose cross-links. Compared with the hydrogels, the low water content of the network resulted in great improvement in mechanical performance because of the cooperative effects given by the reversible catecholato–iron complexes, which contributed to increased cross-link density, and the chain-restricting ionomeric nanodomains. They synthesized a loosely cross-linked amorphous epoxy network with 14 mol % catechol content from monomers of bisepoxide:poly(ethylene glycol) diglycidyl ether, monoepoxide with a triethylsilyl-protected catechol group, and the tetrafunctional diamine cross-linker, as shown in Figure 5a. The protected network was then swollen in acidic aqueous solution to deprotect the catechols by cleaving the triethylsilyl–phenol bonds, and Fe(III) was further introduced to form the catechol–Fe(III) cross-link network (Figure 5b). The process of iron complexation was carefully controlled in order to avoid catechol oxidation. Through small angle and wide angle X-ray scattering, they found that the resulting networks were amorphous without iron precipitates, and that hydrogen-bonded catechol domains were formed in the deprotected network, while catechol–Fe(III) coordinated domains were formed in the iron-treated network. Uniaxial tensile tests revealed significant improvement in stiffness and toughness of the iron-treated networks in comparison with the protected and deprotected ones (Figure 5c). The elastic modulus of the iron-treated network reached 184 ± 14 MPa, which is 770-fold higher than the protected networks. The strength (21.9 ± 0.8 MPa) increased 58-fold, whereas the tensile toughness (22 ± 2 MJ/m^3^) increased 92-fold over the protected materials. These improvements resulted from the considerable increases in the energy dissociation of catechol–Fe(III) coordination bonds compared with the hydrogen bonds. The mechanical recovery of the iron-treated networks was verified through cyclic tensile tests and stress relaxation (Figure 5d). These showed that the iron-treated networks have good recovery at small applied strains (ε ≤ 30%) because the unbroken coordination bonds and covalent network can preserve shape-memory and drive the stretched network back to the original state entropically. However, when the applied strain is larger than 50%, the breaking of coordination bonds can only reform at newly accessible sites, which induces a residual strain and in turn results in stress-free network chains (Figure 5e). This combination of the bioinspired dynamic catechol–Fe(III) coordination bonds with covalent bonds of a cross-linked epoxy network in a dry environment could open up a new avenue for self-healing materials with enhanced mechanical performance.

**Figure 6:**
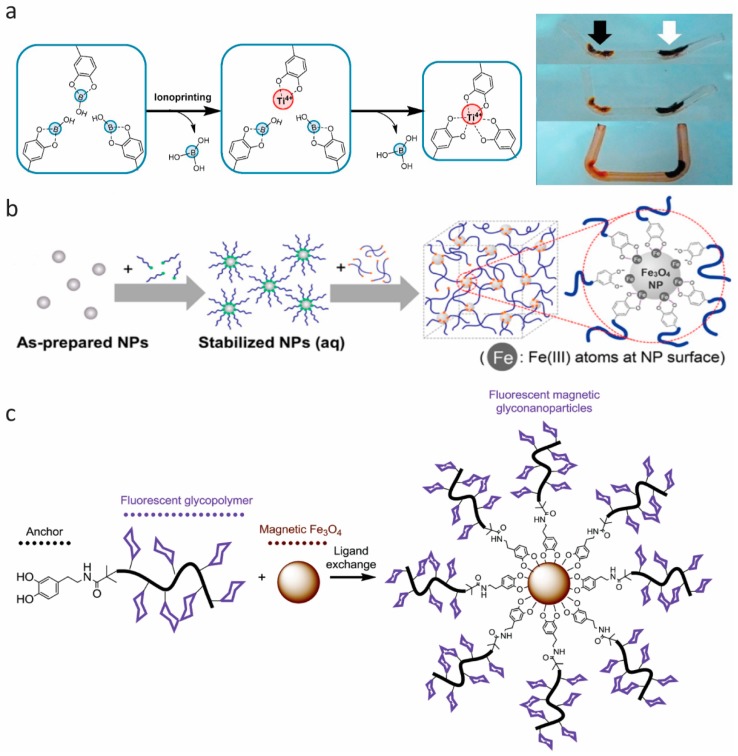
Multifunctional materials. (**a**) Schematic representation of the interaction of –Ti(IV)–catechol in the ionoprinted region, and time lapse photographs showing the actuation of the ionoprinted hydrogel. From top to bottom: The actuation of a hydrogel ionoprinted with titanium (15 V, black arrow) and iron (5 V, white arrow) electrodes, after incubation in pH 9.5, 0.1 M NaCl for 4 min, and after incubation in ethanol for 30 min. Reprinted from [74], Copyright 2016, with permission from Elsevier. (**b**) Schematic illustration of Fe_3_O_4_ nanoparticle (NP) cross-linked hydrogel, with stabilized Fe_3_O_4_ nanoparticles incorporated into 4-arm catechol-terminated poly(ethylene glycol). Reprinted (adapted) with permission from [75]. Copyright 2016 American Chemical Society (**c**) Schematic illustration of magnetic Fe_3_O_4_ nanoparticle with dopamine-terminal fluorescent glycopolymers. Reproduced from [76] with permission of The Royal Society of Chemistry.

Besides hydrogels and elastomers, the self-healing properties of catechol-containing polymers also work under solid-state conditions, such as thin films [64,65,71,72] and aerogels [73]. Moreover, many researchers have been working on the multifunctionality of self-healing adhesives based on metal–catechol coordination. In 2016, Lee et al. [74] applied the catechol–metal coordination chemistry in a hydrogel actuator (Figure 6a), which arose from the bending of the gels at an ionoprinting site due to a severe cross-linking density gradient. Based on this gradient, they modulated the movement of a hydrogel actuator with various metal ions (copper, zinc, aluminum, and titanium) [74]. The group of Holten-Andersen engineered self-healing materials by incorporating iron oxide nanoparticles into a catechol-modified polymer, resulting in reversible metal coordination bonds at the polymer–nanoparticle interface. This introduction of magnetic nanoparticles in hydrogel networks may pave the way to designing stimuli-responsive smart hydrogels via remote control (Figure 6b,c) [75,76]. In 2017, Han et al. [77] designed multifunctional, namely magnetic and conductive, self-healable and tissue-adhesive hydrogels, fabricated by grafting polydopamine to nanoparticles (carbon black nanoparticles and Fe_3_O_4_ nanoparticles) and incorporating them into polyacrylamide networks. The successful design of conducive and magnetic hydrogels can be further extended to host other guest nanoparticles for different functions [77]. Huang et al. [66] developed ultrasound-mediated self-healing hydrogels using DOPA-modified PEG-based hydrogels ([PEG–DOPA]_4_–Fe(III)), based on the metal–catechol bonding and its susceptibility to ultrasonic disintegration [66]. Zhao et al. [78] reported a smart underwater adhesive, taking advantage of the adhesive nature of catechol chemistry, host–guest molecular interaction, and responsive polymers. This smart wet adhesive could screen and activate the interfacial interaction by triggering through a local temperature increase.

## 4. DOPA Analogues: Other Polyphenols

Tunable properties are desired characteristics of all kinds of materials. For catechol-based materials, this can be done by modifying the presence of chemical functionalities in the surroundings of the catechol moiety. One way of doing this is to change the nearby environment, as demonstrated by Mu et al. [27] (Section 1.3) and others [34,47,48,50,51]. Another way is to introduce modifications to the catechol-containing molecule itself. The R group (Figure 2a) of DOPA has been subjected to numerous modifications (both existing and synthetic molecules have been explored), usually with the goal of enabling incorporation of the molecule into existing (bio)polymers or to enable direct (co)polymerization of the molecule. Such modifications have recently been thoroughly reviewed by Patil et al. [79].

### 4.1. Ring-Substituted Catechols

Changing the substituents of the aromatic ring in order to dictate, or at least affect, the reaction chemistry of the catechol has been widely explored.

Initially, the biggest interest was in incorporating electron-withdrawing substituents such as –Cl and –NO_2_ on the 4th position (Figure 2b,c) [44,80,81,82,83,84], in order to increase the oxidation potential and decrease p*K*_a_ values of the hydroxyl groups, thus stabilizing the catechol form and shifting increased coordination stoichiometry towards lower pH [25,82,83]. Later, a broad range of substitutions of the aromatic ring has been explored (Figure 2) [85].

Recently, the 4-nitro catechols have been incorporated in various material designs, both utilizing coordination to Fe(III) ions to make tough, yet injectable and healable plastics [60] and coordination to Fe_3_O_4_ to yield light, self-healable, magnetic networks [86]. Additionally, materials based on 4-nitro catechol–boronates have been used as cross-linkers in polymeric adhesives [87] and to direct self-assembly of block polymers into micelles [88,89]. Self-polymerization of 4-nitro catechols [65] and 4-methoxy catechols [90] (Figure 2b,g) to alternative polydopamine materials with photocatalytic activities and accelerated oxidation (polymerization), respectively, has been developed. Several dopamine derivatives (Figure 2a,b,d–f,h,i, R = CH_2_CH_2_NH_2_) were synthesized by Rote et al. [85] in 2017, and the ^1^H nuclear magnetic resonance (NMR) chemical shift of the aromatic proton in the meta position to the substituent was found to correlate with the Hammett σ_m_ parameter [91] in the order: Cyclic < OH < CO_2_H < Br < CN < NO_2_ (e < i < h < d < f < b in Figure 2, respectively). This is in agreement with expectations from the electronegativity of the substituents, and demonstrates that substitutions of the aromatic ring of catechols impact the chemical behavior of the surrounding hydrogen atoms.

Furthermore, 4-bromo catechols (Figure 2d) and pyrogallols (Figure 2j) have been tested for use in polymeric antioxidants [92,93]. In general, extensive studies on pyrogallolic species (also called tannins) have been developed during recent years for countless applications. Such materials have recently been reviewed by Rahim et al. [16] and Ejima et al. [12]. In brief, several new materials based on metal–pyrogallol coordination have been developed, including new polymer and hydrogel materials [94,95,96], functional coatings and films [97,98,99,100,101], functional nanocomposite materials [102,103,104,105,106], nanoparticles and capsules [107,108], and even solar cell dyes [109,110].

**Figure 7:**
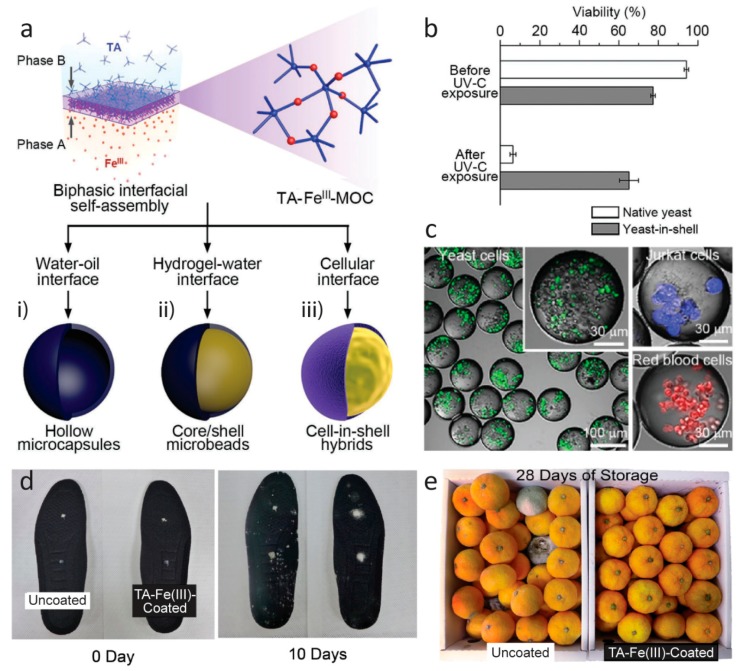
(**a**) Schematic representation of interfacial supramolecular self-assembly of Fe(III) and tannic acid (TA) at various interfaces. Fe(III) ions are preloaded into one phase (phase A), and the supramolecular self-assembly is induced at the interface of two immiscible phases by TA in the other phase (phase B). (**b**) Cytoprotective capability of yeast-in-shell hybrids (obtained from path (**a**,iii)) from ultraviolet (UV)-C irradiation. After fluorescein diacetate treatment, the cells in green were considered alive. All data are shown as the mean ± standard deviation (*n* = 3). (**c**) Confocal laser scanning microscopy images of the microcapsules (obtained from path (**a**,i)) that encapsulate yeast cells (green), Jurkat cells (blue), or red blood cells (red). The cells were labeled with CellTracker^TM^ green CMFDA (5-chloromethylfluorescein diacetate) dye (for yeast cells), CellTracker^TM^ violet BMQC dye (for Jurkat cells), and CellTracker^TM^ deep red dye (for red blood cells). (**a**–**c**) Reproduced with permission from [100]. Copyright 2017 WILEY-VCH Verlag GmbH & Co. KGaA, Weinheim. (**d**) Optical photographs of uncoated and TA–Fe(III)-coated shoe insoles after inoculation of *Trichophyton rubrum* (white) and (**e**) mandarin oranges (*Citrus reticulata*) after storage at room temperature. Adapted from [113], CC BY 4.0 [115].

Especially, the use of metal-coordinated TA (Figure 2k), a poly-pyrogallol molecule derived from natural materials [111,112], shows promising results. The coatings have recently been proved useful in the field of food preservation [102,113] and the groups of Choi [100,114] and Caruso [98] have published new results on TA–Fe(III) for encapsulation, following their sporulation-inspired research in TA–Fe(III) for encapsulation of cells [101]. Figure 7 displays recent work from Choi and co-workers on TA–Fe(III) encapsulation/coating by different strategies and on different length scales. An interface diffusion approach to encapsulation of various substrates was used for different biologically relevant applications [100], while spray-on nanocoating afforded coatings with antimicrobial properties proved useful for the preservation of food [113].

### 4.2. Ring Modification: Pyridinone Catechols

**Figure 8:**
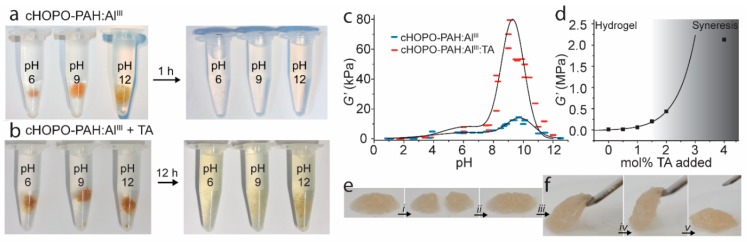
(**a**,**b**) Hydrogel reversibility: Competitive binding experiments with 150 mM ethylenediaminetetraacetic acid (EDTA) (aq, pH 5) on (**a**) cHOPO–PAH:Al(III) and (**b**) cHOPO–PAH:Al(III) + TA (1 mol %) hydrogels with pH 6, 9, and 12. (**c**,**d**) Hydrogel stiffness: Storage modulus at an angular frequency of 1 s^−1^ and strain amplitude of 2%: (**c**) cHOPO–PAH:Al(III) (blue) and cHOPO–PAH:Al(III) + TA (1 mol %) (red) hydrogels. The widths of the symbols represent the estimated standard uncertainty of the pH values. The lines serve as guides to the eye. (**d**) cHOPO–PAH:Al(III) + TA hydrogels at pH 9 with varying amount of TA added. The curve is an exponential fit to the data points before syneresis sets in. (**e**,**f**) Qualitative self-healing test of hydrogel at pH 9: (**e**) The hydrogels are cut (i), allowed to recover (ii), and (**f**) subsequently lifted and strained (iii–v). Figure adapted from reference [117].

Instead of using substitutions on the catechol, the aromatic ring itself can also be changed to great effect. Such a catechol analogue, where the ring itself is changed to a pyridinone, was for the first time incorporated in a mussel-inspired hydrogel construct in 2013 by Menyo et al. [116]. Here, the metal coordination capabilities 2-methoxy-3-hydroxy-4-pyridinone (HOPO) (Figure 2l) were compared to DOPA and 4-nitro catechols in PEG-based hydrogels. As HOPO has significantly lower phenolic p*K*_a_ values, metal coordination was favored at lower pH as expected. Furthermore, the authors concluded that in contrast to the unmodified catechol, the catechol of HOPO is resistant to oxidation by oxygen (ageing), pH, and redox reactions with Fe(III)→Fe(II). In an follow-up study in 2015, the same authors demonstrated the capability to achieve rate-dependent stiffness and recovery in interpenetrating network hydrogels of loosely covalently cross-linked poly(hydroxyethyl acrylamide) and metal-coordinated HOPO–PEG [116]. Utilizing the oxidation resistant nature of HOPO, we further developed a double cross-linked network hydrogel system with pH-dependent and self-healing properties and tunable stiffness by controlling the amount of oxidation-induced (covalent, by TA) and coordination-based cross-linking of 1-(2′carboxyethyl)-2-methyl-3-hydroxy-4(1H)-pyridinone-grafted polyallylamine (cHOPO–PAH) (Figure 8) [117].

This work demonstrates how the HOPO coordination in an amine-rich environment can ensure strong, yet reversible cross-linking even at low pH due to lowered phenolic p*K*_a_ values and at high pH due to the oxidation resistance of HOPO. The use of a pyridinone ring means that the doubly deprotonated HOPO only has a charge of −1. Therefore, the tris-complex with trivalent metal ions is in fact charge neutral, alleviating the need for counter ions to ensure local charge neutralization. We suggest that this feature may be valuable in many other systems as a handle on charge balance and entropic effects in supramolecular assembly. Addition of oxidizable catechols in the form of TA to the Al(III) cross-linked cHOPO–PAH hydrogels allowed significant increase in the stiffness of the gels by introducing small amounts of covalent cross-links (between TA and the amine on the polymer network). As a result of the oxidation resistance of HOPO, this stiffening was achieved without loss of self-healing properties due to sacrificial rupture of the reversible coordination cross-links [116,117]. This work shows how oxidation control of catechols significantly modulates their performance in materials.

## 5. Summary and Outlook

The versatile chemistry of polyphenols enables their use in smart and functional materials for many purposes. This has led to a considerable number of publications utilizing this versatility by incorporating naturally occurring polyphenols into many types of materials, including hydrogels, capsules, coatings, and nanoparticles for purposes within the fields of biomedical, food, and materials science. With the goal to gain a better understanding of the mechanisms and regulation used by biology, and to improve and specify the design of materials for different purposes, the field has expanded: Chemical modification of polyphenols has proven to be an effective tool for regulating the acidic, oxidative, and binding properties of the catechol moiety. Additionally, studies of the impact of the chemical environment of the polyphenol moiety reveal new information on how to modulate its chemistry. This sets the stage for widespread use of polyphenol/metal complexes in materials research.

We expect that the reversible metal coordination chemistry will develop further, both as a basic assembly mechanism and as a means to provide rapid self-healing responses. Even with Fe(III), hydrogels were found to be well tolerated in biocompatibility assays [51], indicating that the potential cytotoxicity of some metal ions is reined in by the strong coordination. This suggests that such materials may find use in biomaterials settings. With the generalizations to other materials than hydrogels, cf. elastomers in Section 3 and polyphenol coatings in Section 4, the door is open to a wide range of possible applications from food technologies to bulk materials. In this context, further developments of catechol analogues and the incorporation of such analogues in materials seem especially important, as these allow for controlling the reactivity of the molecules, their pH dependence, and the redox behavior; and thus affords better control over reaction conditions and covalent oxidative coupling that can even be removed completely to preserve long-term self-healing capabilities at all pH values (Section 4). The combination with other modes of self-healing (e.g., histidinato–copper complexes [19]) may afford materials with multiple relaxation modes [118], providing excellent control over self-healing capabilities. Metal coordination thus emerges as a highly promising tool for design and synthesis of advanced self-healing materials.
